# UNDERSTANDING ACTIVITY ENGAGEMENT EXPERIENCE AMONG RESIDENTS WITH DEMENTIA IN LONG-TERM CARE HOMES

**DOI:** 10.1093/geroni/igae098.2043

**Published:** 2024-12-31

**Authors:** Ziying Zhang, Habib Chaudhury

**Affiliations:** Simon Fraser University, Vancouver, British Columbia, Canada; Simon Fraser University, Vancouver, British Columbia, Canada

## Abstract

Recreational and meaningful activities in long-term care homes are crucial in promoting well-being among residents with dementia. While existing research primarily focuses on planned activities facilitated by staff, little attention has been given to self-directed activities initiated by residents themselves, reflecting their intrinsic motivation, lifestyle, and lifelong interests such as walking, gardening, meal preparation and other household activities. In this study, activity is defined as personally meaningful behavior, including both programmed and self-directed activities, and supported by social and/or physical environmental stimuli. Utilizing critical ethnography in a long-term care home in Canada, our research aims to: 1) gather descriptive data on the types, diversity and process of residents’ activity engagement, and 2) examine the care home setting in its natural context to uncover the social practices, built environmental features and underlying organizational discourses that influence activity engagement. Observations will focus on residents’ engagement in various activities and their interactions with staff and the physical environment. Interviews will be conducted with activity staff and non-activity staff (e.g., care aides, nurses) to explore their perspectives on activity engagement, person-centered approach, and their respective roles in promoting engagement. This study offers insights into residents’ experiences with both structured and self-directed activities, and the influence of the care home’s social and physical environments on engagement. The study addresses the knowledge gap in meaningful engagement through self-directed activities and the role of social and physical environments. Fieldwork will be completed in summer and preliminary findings based on interviews and initial observations will be presented.

